# Blood Volume as a new functional image-based biomarker of progression in metastatic renal cell carcinoma

**DOI:** 10.1038/s41598-021-99122-1

**Published:** 2021-10-04

**Authors:** Aska Drljevic-Nielsen, Finn Rasmussen, Jill Rachel Mains, Kennet Thorup, Frede Donskov

**Affiliations:** 1 Department of Radiology, Aarhus University Hospital, Aarhus N, Denmark; 2 Department of Oncology, Aarhus University Hospital, Aarhus N, Denmark

**Keywords:** Cancer imaging, Tumour angiogenesis, Tumour biomarkers, Renal cancer

## Abstract

RECIST v1.1 has limitations in evaluating progression. We assessed Dynamic Constrast Enhanced Computed Tomography (DCE-CT) identified Blood Volume (BV) for the evaluation of progressive disease (PD) in patients with metastatic renal cell carcinoma (mRCC). BV was quantified prospectively at baseline, after one month, then every three months until PD. Relative changes (ΔBV) were assessed at each timepoint compared with baseline values. The primary endpoint was Time to PD (TTP), the secondary endpoint was Time to the scan prior to PD (PDminus1). Cox proportional hazard models adjusted ΔBV for treatments and International mRCC Database Consortium factors. A total of 62 patients had analyzable scans at the PD timepoint. Median BV was 23.92 mL × 100 g^−1^ (range 4.40–399.04) at PD and 26.39 mL × 100 g^−1^ (range 8.70–77.44) at PDminus1. In the final multivariate analysis higher ΔBV was statistically significantly associated with shorter Time to PD, HR 1.11 (95% CI 1.07–1.15, *P* < 0.001). Also assessed at PDminus1, higher ΔBV was significantly associated with shorter time to PD, HR 1.14 (95% CI 1.01–1.28, *P* = 0.031). In conclusion, DCE-CT identified BV is a new image-based biomarker of therapy progression in patients with mRCC.

## Introduction

The rapid development of new therapies for metastatic renal cell carcinoma (mRCC), such as angiogenesis inhibitors and immune check-point inhibitors, has not been accompanied by a corresponding development in the response evaluation criteria^1^. The current gold standard for evaluation of treatment response is the Response Evaluation Criteria in Solid Tumors version 1.1 (RECIST v1.1)^2,3^, which provides only morphological information, while not taking into account physiological and biological changes within the tumor^2,4,5^.

RECIST v1.1 defined progression (PD) is characterized by an increase > 20% in the sum of unidimensional diameters in up to 5 target lesions or the appearance of a new lesion^2,3^. However, immune checkpoint inhibitors may lead to immune cell infiltration in the tumor tissue, resulting in inflammatory swelling, i.e., the tumor lesion increases in size or new lesions appear as part of pseudo-progression^5^. Angiogenesis inhibitors can induce tumor necrosis leading to an increase in size of the tumor^6^. Therefore, when monitoring treatment according to RECIST v1.1, an enlargement in the tumor can lead to interpretational distress over stopping therapy, as the patient may not necessarily have treatment failure. The clinical dilemma is real. Stopping therapy too early without unequivocal progression means abandoning efficient therapy; stopping therapy too late may be fatal, as approximately half of patients are lost to further treatment at the time of progression^7–9^.

Dynamic contrast-enhanced computed tomography (DCE-CT) is a functional imaging modality consisting of repeated scans over a single target lesion (primary tumor or metastases) enabling an assessment of changes in contrast enhancement of the scanned tissue. Using advanced software techniques, these data can be used to calculate functional parameters, such as blood volume (BV) using histogram analysis. This parameter correlates to vascularity and provides additional information to the morphological information obtained from the routine Contrast-Enhanced CT (CE-CT)^10–12^.

DCT-CT identified BV at baseline was recently identified as a new, independent prognostic factor in patients with metastatic renal cell carcinoma (mRCC), that may add to the prognostic accuracy of International Metastatic renal cell carcinoma Database Consortium (IMDC) criteria^13,14^. Early decline in DCE-CT parameters have shown to predicted treatment response and a favorable outcome in patients with mRCC treated with immunotherapy and targeted therapies^15–17^. Even though it is well established that a high baseline BV was an independent biomarker for favorable survival outcome^13^, it remains to be assessed whether BV can be used as a biomarker for progression during treatment in patients with mRCC.

The aim of the study was to examine whether BV, identified by DCE-CT, could be used as an image-based biomarker of therapy progression in patients with mRCC.

## Materials and methods

### Patients

Patients with biopsy verified mRCC treated at Aarhus University Hospital, Denmark, were included from two prospective studies: the Angiogenesis Inhibitor Study (AIS) and the Danish Renal Cancer Group Study-1 (DaRenCa-1). Patients in the cohort study AIS (N = 33) were enrolled between January 2012 and September 2016 and were treated with first-line pazopanib (N = 12) (Votrient: Novartis Oncology, before 2015: GlaxoSmithKline), sunitinib (N = 12) (Sutent: Pfizer), or temsirolimus (N = 9) (Torisel: Pfizer Inc). The randomized phase II clinical trial DaRenCa-1 (N = 89) compared the effect of subcutaneously administered Interleukin 2 (IL-2) (aldesleukin, Proleukin: Novartis Vaccines and Diagnostics) and interferon alpha (IFN-α) (IFN-α2b, IntronA: Merck) with or without intravenously administered bevacizumab (N = 45 and N = 44, respectivly) (Avastin: Genentech, Roche) in patients enrolled between October 2009 and November 2014^18^. Treatment in both AIS and DaRenCa-1 cohorts was given until RECIST v1.1-defined progression.

The inclusion criteria for the DaRenCa Study-1 were: histologically verified clear cell mRCC, no prior oncologic treatment, measurable metastatic disease according to the RECIST v.1.1 criteria, favorable or intermediate risk MSKCC group, Karnofsky Performance Status ≥ 70% and adequate kidney function (serum creatinine < 150 micromol/L). The inclusion criteria for AIS were: histologically verified mRCC, no prior oncologic treatment and adequate kidney function (estimated glomerular filtration rate (eGFR < 35 ml/min).

Using the same cohort of patients as in this current study, the association between baseline BV and survival outcome adjusted for baseline features^13^ and the association between DCT-CT parameters and early treatment response have been published^15–17^.

Approval by the Central Denmark Region Ethics Committee and The Central Denmark Data Protection Agency was granted and written informed consent was obtained before inclusion started. The study was performed in accordance to the approved guidelines by the Central Denmark Region Ethics Committee and The Central Denmark Data Protection Agency. DaRenCa-1 was registered at ClinicalTrials.gov (identifier NCT01274273) and approved by the Danish Medicines Agency.

Electronic medicinal charts were used to retrieve information about baseline clinical factors, treatments and baseline IMDC prognostic factors^14^.

**Figure 1:**
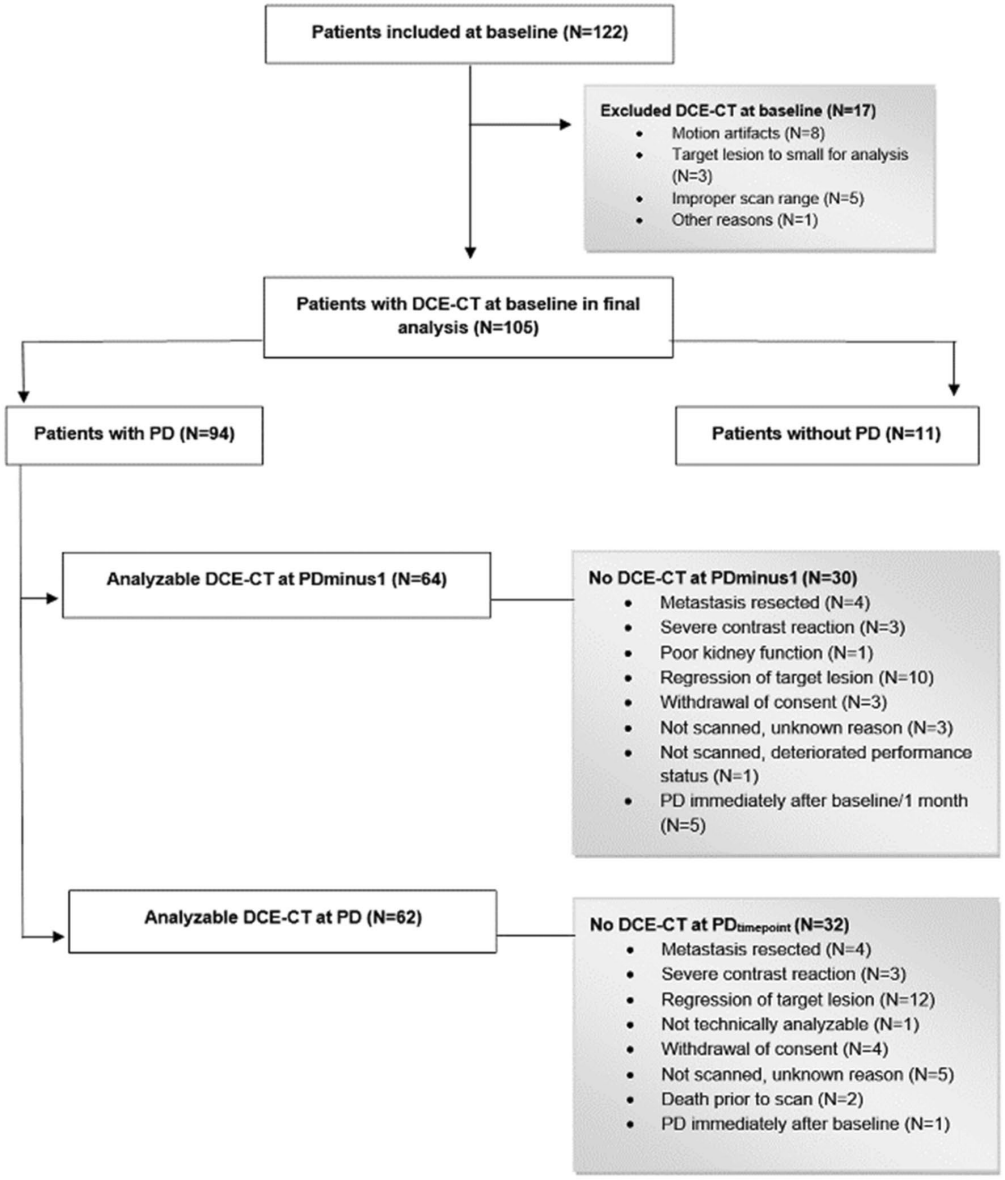
Flowchart of prospectively included patients with mRCC. Patients had Dynamic Constrast Enhanced Computed Tomography (DCE-CT) at baseline before initiation of therapy, and until progressive disease (PD) or the scan prior to PD (PDminus1). At baseline 105 patients had analyzable scans. Main reasons for not having an analyzable scan at PD time point, or PDminus1 time point, were lack of PD, complete regression of the target lesion, or surgical resection of the target lesion.

A total of 105 patients (DaRenCa-1, N = 76 and AIS, N = 29) and 483 analyzable DCE-CT scans were included in the study, where 62 and 64 patients had an analysable DCE-CT scan at PD and the scan prior to PD (PDminus1), respectively, Fig. 1.

### CE-CT and DCE-CT

A routine contrast enhanced (CE) CT was performed at baseline and every 3 months until progression and was assessed according to RECIST 1.1^2,3^. All clinical decisions were based on routine CE-CT scan results.

DCE-CT was performed at baseline, after 1 month of therapy and every three months until progression. Initially, a DCE-CT scan of a single target lesion was performed, followed by a routine CE-CT scan of the thorax, abdomen, and pelvis. Patients remained supine for 10 min between the scans. Based on prespecified protocol criteria, an experienced radiologist selected a representative target lesion that was optimal for functional imaging. The target lesions scanned with DCE-CT were located in the lung (N = 21), pleura (N = 5), supraclavicular/thoracic lymph nodes (N = 16), retroperitoneal lymph nodes (N = 8), kidney (N = 14), kidney bed (N = 6), adrenal gland (N = 3), bone (N = 12), liver (N = 8), pancreas (N = 7), extra/intra abdominal soft tissue (N = 3 and N = 2, respectively); the same DCE-CT technique was used irrespectively of target lesion location.

Before each DCE-CT scan, 60 ml iodixanol (Visipaque, GE Healthcare) 270 mg I/mL at 6 mL/s was administered intravenously. Before each routine CE-CT, iodixanol (Visipaque, GE Healthcare) 270 mg I/ml based on body weight (maximum 180 ml) at 4 mL/s was administered intravenously. In the event of minor reactions to iodixanol, patients were subsequently given iohexol (Omnipaque, GE Healthcare) 300 mg I/mL instead.

DCE-CT and CE-CT scans were performed using either Philips Brilliance 64 or iCT 256 (Philips Healthcare). DCE-CT consisted of 2-s scan cycles for a total of 70 s; median z-axis of 8 cm (range, 4–14.5 cm), median mAs of 100mAs (range, 100–210 mAs), and median kVp of 100 kVp (range, 80–180 kVp). The median dose length product was 1050.10 mGy x cm (range, 186.9–3009.00 mGy x cm) and the median computed tomography dose index was 131.29 mGy (range, 18.80–296.27 mGy).

Routine CE-CT scans were obtained using attenuation based current modulation; 120 kVp of peak voltage, 0.75 s of rotation time, a collimation of 64/128 × 0.625 mm, and a pitch of 0.925.

### 4D imaging analysis

The prototype software program Advanced Perfusion and Permeability Application, Philips (Philips, Healthcare) was used to analyze DCE-CT data in four dimensions (4D). After loading the dynamic data, a spatial filtration and motion correction was performed using a non-rigid registration. The software program used the deconvolution method^19^ to calculate BV (mL × 100 g^−1^) and display corresponding BV maps.

Data were then loaded into Intellispace 6.0 Multimodality Tumor Tracking (Philips, Healthcare). A semi-quantitative three dimensional (3D) sculpt-tool was used to delineate the target lesion as the Volume of Interest (VOI) using the morphological DCE-CT images at arterial peak enhancement. When the 3D analysis was combined with the time dimension in DCE-CT due to repeated measurements, it resulted in a 4D analysis. A radiologist, blinded to the treatment group and survival outcomes, performed all the analyses. This particular method has previously shown excellent interobserver correlations^17^.

MATLAB (v. R2015b, MathWorks Inc.) was used to analyze the dynamic BV data based on the VOI on DCE-CT images at peak arterial enhancement. Histogram values of BV were extracted based on the DCE-CT VOI using in-house programmed scripts. The median values for BV was calculated for each histogram and used for assessment, as this value previously has shown the best correlation with patient outcome in mRCC^17^.

### Statistical analysis

Compared to baseline, relative changes in BV were calculated in percent (%) at each scan timepoint (X_timepoint_) until RECIST v1.1 defined PD:

$$\frac{{\left( {\left[ {{\text{X}}_{{{\text{timepoint}}}} } \right] - \left[ {{\text{Baseline}}} \right]} \right)}}{{\left[ {{\text{Baseline}}} \right]}}*100$$

The association between baseline factors and Time to progression (TTP), defined as the time between baseline and the scan timepoint of RECIST v1.1 defined PD or cancer related death, whichever came first, was examined using a univariate Cox proportional hazards models expressed as hazards ratios (HR) with 95% confidence intervals (CI). Baseline univariate factors, including individual IMDC risk factors, prior nephrectomy, age and gender, with *P* < 0.10 and treatment groups were included in the multivariate Cox proportional hazards models.

ΔBV was assessed as continuous variables presented as 20-percent point increasements at each timepoint. Univariate and multivariate Cox proportional hazards models were used to examine the association between ΔBV and PD using two different endpoints. The primary endpoint was TTP and the secondary endpoint was Time to the scan prior to PD (PDminus1), i.e. to assess if PD could be detected on the scan one timepoint earlier. For patients experiencing cancer related death or PD due to clinical evaluation or supplementary imaging, a consensus was made regarding classification of the latest DCE-CT scan being either PD (N = 9) or PDminus1 (N = 4) events.

The effect between treatment groups and ΔBV were examined by constricting the multivariate analyses to the patients treated with angiogenesis inhibitors and the immunotherapy, respectively. The proportional hazards assumptions were tested graphically by Schoenfeld residuals against the time and were fulfilled.

The median follow-up time in alive patients was assessed using the reverse Kaplan–Meier survival curves. A Receiver Operating Characteristic (ROC) analysis was performed to identify a possible cut-off at PD and PDminus1 for the relative change in the continuous DCE-CT parameters that were deemed significant in the multivariate Cox regression analysis. The ROC generated area under the curve (AUC) < 0.8 was considered not to have predictive value and was therefore not eligible for estimating a cut-off value^20^. All tests were two-sided and P values below 0.05 were considered as statistically significant. IBM SPSS Statistics for Windows (Version 27.0, IBM Corp.) was used to perform all statistical analyses.

## Results

### Patients

Baseline patient characteristics are presented in Table 1.

**Table 1:** Baseline characteristics.

Factor	N (%)
Total	105 (100)
Gender	
Male	77 (73.3)
Female	28 (26.7)
IMDC group	
Favorable	22 (21.0)
Intermediate	56 (53.3)
Poor	27 (25.7)
Karnofsky Performance status < 80%	5 (4.8)
Interval between RCC diagnosis and 1st line therapy < 1 year	77 (73.3)
Hemoglobin < LLN	57 (54.3)
Neutrophils > ULN	16 (15.2)
Trombocytes > ULN	24 (22.9)
Albumin corrected calcium > ULN	11 (10.5)
Histology	
Clear cell	98 (93.3)
Non clear cell	7 (6.7)
Prior nephrectomy	
Yes	87 (82.9)
No	18 (17.1)
Synchronous metastatic disease	
Yes	60 (57.1)
No	45 (42.9)
Sarcomatoid differentiation	
Yes	16 (15.2)
No	89 (84.8)
Treatment	
IL-2 and IFN-α	37 (35.3)
IL-2, IFN-α and bevacizumab	39 (37.1)
Sunitinib, pazopanib or temsirolimus	29 (27.6)

*ULN* Upper Limit of Normal, *LLN* Lower Limit of Normal, *IL-2* Interleukin-2, *IFN-α* interferon alpha, *IMDC* International Metastatic Renal Cell Carcinoma Consortium.

At the time of primary diagnosis approximately half of the included patients had T1-T2 disease (48%), while the other half had T3-T4 disease (51%). Negative nodal status (N0) was seen in 57 patients (54%), while 25 patients (24%) had a positive nodal status (N1) and 23 patients (22%) had an unknown nodal status (NX). A total of 6 patients (6%) had a history of previous cancer five years prior to the mRCC diagnosis (basal cell carcinoma (N = 4), prostate adenocarcinoma (N = 1) and caecum adenocarcinoma (N = 1)).

All included patients were naive to systemic oncological treatment and received 1st line oncological treatment. Most patients had a prior nephrectomy (83%), 16 patients (15%) had prior excision of a metastasis and 4 patients (4%) had prior radiotherapy.The majority were male (73%), had clear cell histology (93%), and 57% had synchrounous cancer (presence of metastastatic disease ≤ 3 months of initial cancer diagnosis); 15% had sarcomatoid differentiation. According to the IMDC criteria, 21% were favorable, 53% intermediate, and 26% poor prognostic category, Table 1.

The median Time to PD was 9.15 months (95% CI 6.87–11.42) and the median follow-up in alive patients was 66.07 months (95% CI 32.36 – 99.78). The baseline target lesion volume was at median 18.32 cm^3^ (range 0.90–572.10) and baseline median BV was 32.87 mL × 100 g^−1^ (range 9.52–92.87).

Baseline anemia, HR 1.56 (95% CI 1.04–2.36, *P* = 0.033) and baseline neutrophilia, HR 2.89 (95% CI 1.65–5.08, *P* < 0.001) were associated with shorter time to progression, Table 2.

**Table 2:** Univariate association of baseline characteristics with time to progression.

Factor	TTP	
	HR (95% CI)	*P*
Individual IMDC risk factors:		
Karnofsky Performance status < 80%	2.49 (1.00;6.21)	0.051
Interval between RCC diagnosis and 1st line therapy < 1 year	1.61 (1.00;2.59)	0.052
Hemoglobin < LLN	1.56 (1.04;2.36)	***0.033***
Neutrophils > ULN	2.89 (1.65;5.08)	** < 0.001**
Trombocytes > ULN	1.51 (0.95;2.41)	0.081
Albumin corrected calcium > ULN	1.22 (0.63;2.35)	0.556
Prior nephrectomy	0.82 (0.48;1.42)	0.480
Female gender	1.43 (0.91;2.27)	0.122
Age above median (> 60.1 years)	0.72 (0.48;1.09)	0.118
Treatmentgroup		0.288
IFN-α and IL-2	Reference	
IFN-α, IL-2 and bevacizumab	0.65 (0.39;1.11)	
Sunitinib, pazopanib or temsirolimus	0.81 (0.51;1.30)	

*TTP* Time to progression, *ULN* Upper Limit of Normal, *LLN* Lower Limit of Normal, *IL-2* Interleukin-2, *IFN-α* interferon alpha.

### Univariate analyses of relative changes in BV

Median BV at PD was 23.92 mL × 100 g^−1^ (range 4.40–399.04) and was 26.39 mL × 100 g^−1^ (range 8.70–77.44) at PDminus1. In univariate analysis, a higher ΔBV was associated with shorter time to PD, HR 1.11 (95% CI 1.07–1.15, *P* < 0.001).

### Multivariate analysis of relative changes in BV

**Figure 2:**
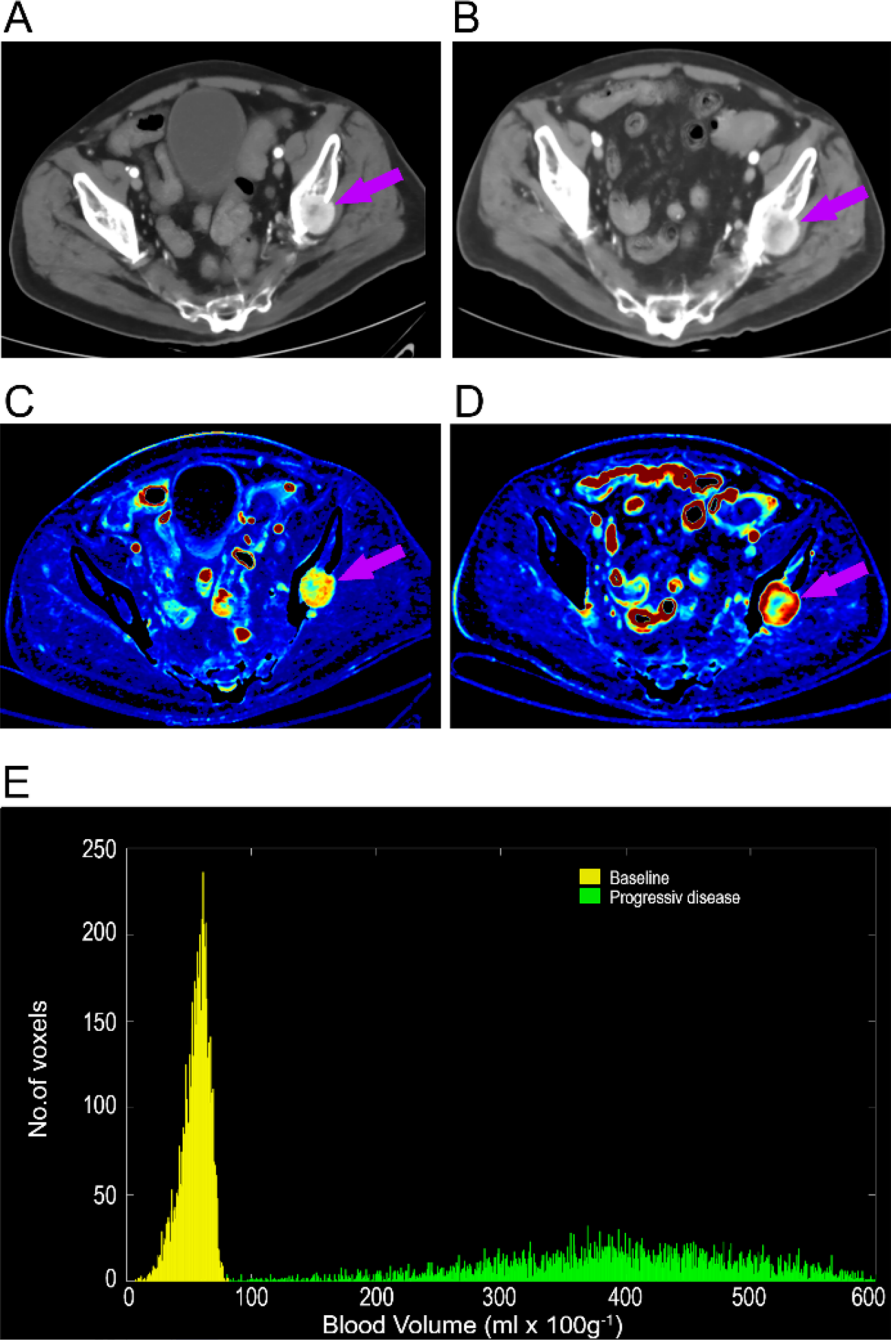
A patients with mRCC and a left iliac bone metastasis (purple arrow) on a routine CE-CT (**A**) at baseline and (**B**) at PD; and DCE-CT (**C**) at baseline and (**D**) at PD. (**E**) The BV histograms of the metastasis depicts an increase in median BV values from baseline (57.29 mL × 100 g^−1^) to PD (399.04 mL × 100 g^−1^). *CE-CT* contrast enhanced Computed-Tomography, *DCE-CT* dynamic Contrast enhanced Computed-Tomography, *PD* progressive disease.

In the final multivariate analysis, higher ΔBV were independently associated with a shorter time to PD, HR 1.11 (95% CI: 1.07–1.15, *P* < 0.001), Fig. 2 and Table 3.

**Table 3:** Multivariat association at PD.

Factor	Time to PD	
	HR (95% CI)	*P*
Δ Blood volume	1.11 (1.07;1.15)	< 0.001
Interval between RCC diagnosis and 1st line therapy < 1 year	1.81 (1.10;2.96)	0.018
Neutrophils > ULN	2.46 (1.10;5.48)	0.026
Treatment group		0.040
IFN-α and IL-2	Reference	
IFN-α, IL-2 and bevacizumab	0.96 (0.58;1.56)	
Sunitinib, pazopanib or temsirolimus	0.44 (0.23;0.86)	

The table exhibits factors that remained significant in the final multivariate analysis.

*PD* progressive disease, *IL-2* Interleukin-2, *IFN-α* interferon alpha.

**Figure 3:**
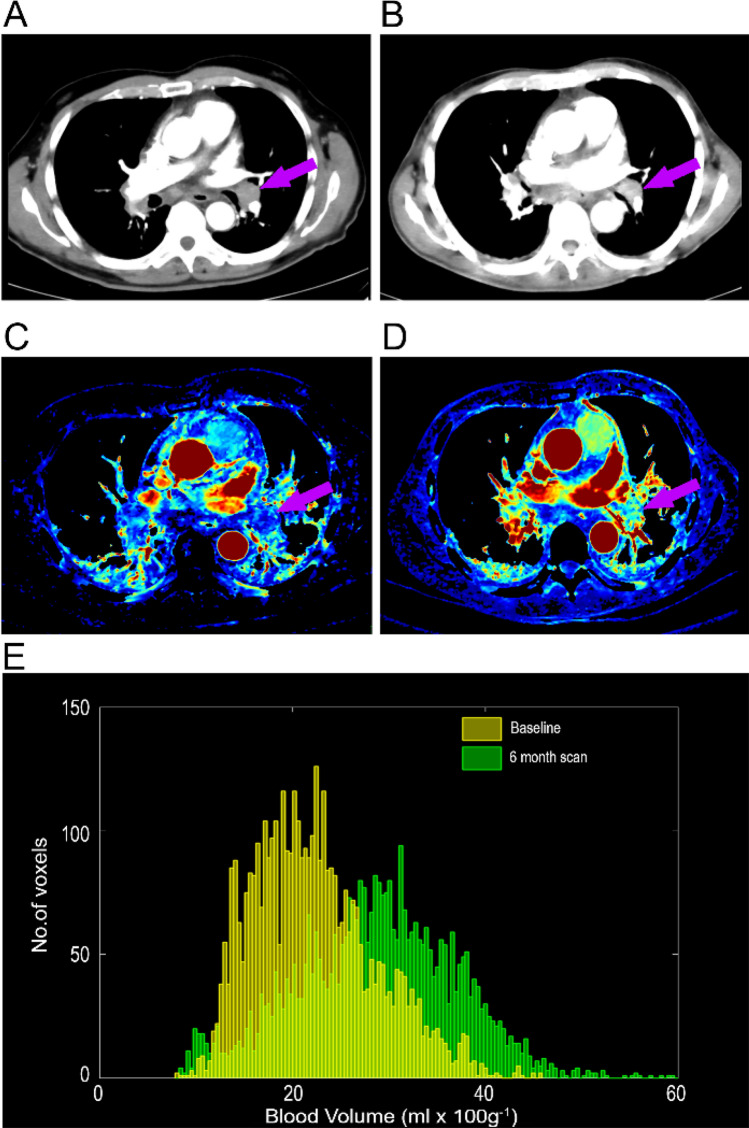
A patients with mRCC and a bronchopulmonary metastasis (purple arrow) on a routine CE-CT (**A**) at baseline and (**B**) the scan prior to progression, PDminus1; and DCE-CT (**C**) at baseline and (**D**) at PD. (**E**) The BV histograms of the metastasis depicts an increase in median BV values from baseline (21,57 mL × 100 g^−1^) to PDminus1 (28,85 mL × 100 g^−1^). *CE-CT* contrast enhanced Computed-Tomography, *DCE-CT* dynamic Contrast enhanced Computed-Tomography, *PDminus1* scan prior to progressive disease.

Also assessed at PDminus1 an higher Δ BV were independently associated with shorter time to PD, HR 1.14 (95% CI 1.01–1.28, *P* = 0.031), Fig. 3 and Table 4.

**Table 4:** Multivariat association at PDminus1.

Factor	Time to PDminus1	
	HR (95% CI)	*P*
Δ Blood volume	1.14 (1.01;1.28)	0.031
Interval between RCC diagnosis and 1st line therapy < 1 year	1.95 (1.19;3.20)	0.007
Treatment group		0.039
IFN-α and IL-2	Reference	
IFN-α, IL-2 and bevacizumab	1.16 (0.75;1.79)	
Sunitinib, pazopanib and temsirolimus	0.52 (0.28;0.97)	

The table exhibits factors that remained significant in the final multivariate analysis.

*PDminus1* Scan prior to progressive Disease, *IL-2* Interleukin-2, *IFN-α* interferon alpha.

Performing the analyses constricted to patients with clear cell RCC, higher ΔBV remained independently associated with a shorter time to PD, HR 1.13 (95% CI: 1.09–1.17, *P* < 0.001) and PDminus1, HR 1.24 (95% CI: 1.10–1.40, *P* < 0.001).

Constricting the multivariate analyses to patients treated with angiogenesis inhibitors, higher ΔBV remained independently associated with a shorter time to PD, HR 1.09 (95% CI: 1.03–1.16, *P* = 0.002), but no association was found at PDminus1. For patients treated with immunotherapy, higher ΔBV remained independently associated with a shorter time to PD, HR 1.15 (95% CI: 1.06–1.25, *P* < 0.001) and PDminus1, HR 1.26 (95% CI: 1.05–1.53, *P* = 0.011), respectively.

### Receiver operating characteristic analysis

AUC for ΔBV was 0.494 (95% CI 0.249–0.694) at PD and 0.468 (95% CI 0.256–0.679) at PDminus1.

## Discussion

This study is the first to demonstrate that higher DCE-CT identified ΔBV is a new image-based biomarker of therapy progression in patients with mRCC. Our results show that patients with a 20%-point higher ΔBV have a 11% higher risk of having RECIST defined PD on the current conventional CT scan, indicating that the relative change in BV can be used as a biomarker to predict progression at the timepoint of RECIST v.1.1 defined progression. Furthermore, we find that patients with a 20%-point higher ΔBV without RECIST v1.1 defined PD on the current conventional CT scan have a 14% higher risk of RECIST v1.1 defined PD on the subsequent scan, suggesting that this biomarker may predict progression at the scan prior to RECISTv.1.1 defined progression. These findings strengthen the utility of BV as a biomarker for progression. Therefore, DCE-CT identified BV may have the potential to be used as a helper to RECIST v1.1 in identifying PD in patients with mRCC and may have the potential to support clinical decision-making during treatment monitoring in mRCC, when RECIST v1.1 is uncertain. However, it was not possible to define a cut off value for BV, limiting the implementation in the clinical setting. Further development in DCE-CT functional imaging in a larger cohort is encouraged, and is needed before it can reach clinical daily life in treatment decision making.

The association between ΔBV and PD was independent of treatment group at the timepoint of RECIST v.1.1 defined progression. However, at the timepoint prior to RECISTv.1.1 defined progression, the association was only significant for patients treated with IL-2 based therapies. These findings could indicate that BV increases more rapidly in patients treated with angiogenesis inhibitor. Further research assessing this matter in a larger cohort is encouraged.

RECIST v1.1 relies solely on morphological information and because changes in tumor size may lag behind pathophysiological changes within the tumor, it is a suboptimal response evaluation tool for patients treated with targeted therapy^21–24^. Characterizing unequivocal progression and changing therapy to the next treatment line at the appropriate time using RECIST v1.1 can be difficult. In the randomized phase III trial comparing pazopanib and sunitinib as first-line therapy in mRCC patients, only half of the patients continued to second-line treatment after PD^8,9^. Pseudoprogression has not been described in the literature on IL-2 immunotherapy and was not observed in this current study. However, pseudoprogression is a challenge during checkpoint immunotherapy and was highlighted in the study of Escudier et al., where the survival rate almost doubled in patients treated with nivolumab beyond RECIST v1.1 defined progression^25^. These studies illustrate and highlight the clinical dilemma of stopping therapy too late or too early.

The increasing need for a better response evaluation tool has led to attempts to improve the RECIST v.1.1 criteria. Choi was the first to combine morphological and functional information measured as CT contrast uptake (Hounsfield unit) in a target lesion on CE-CT. However, the CT contrast uptake was only measured in a single slice making it a major limitation due to intratumoral heterogenicity^26^. The method in this current study evaluated the entire target lesion and thus took into account the intratumoral heterogenicity, making this method superior to the single slice method.

DCE-CT derived BV is a robust parameter, independent of changes in cardiac output, as described by Miles et al.^12^. BV values can be affected by motion and beam hardening artifacts^19^, which can be minimized by instruction in shallow breathing, and by avoiding target lesions close to prosthetics or areas with high concentrations of contrast media agents.

Several studies, focusing on the prognostic significance of DCE-CT parameters at baseline, as well as the evaluation of early treatment response in mRCC, have been conducted^13,15–17,27,28^. The study of Drljevic-Nielsen et al. showed that high baseline BV was a favorable independent prognostic factor for survival outcome^13^, while the studies of Mains et al. showed that early reduction in BV was associated with favorable outcomes, whereas only a smaller reduction or an increase in BV were associated with worse outcomes^15–17^. Our study is the first to demonstrate that a relative higher BV, when compared with baseline, was associated with a higher risk of PD and thus a worse outcome. Summarized, a higher BV at baseline is a favorable feature, whereas an higher ΔBV during treatment is an unfavorable feature.To our knowledge no previous studies have assessed the potential of DCE-CT parameter BV as imaging-biomarker for progression, making our study a first of a kind.

Several limitations to this study must be noted. Firstly, motion artifacts occurred even though patients were instructed in shallow breathing. Secondly, the increased radiation dose in DCE-CT induces a larger stochastic risk for a radiation-induced cancer. However, patients with mRCC have a reduced life expectancy making the risk for radiation-induced cancer very low. Thirdly, of the 105 included patients at baseline, ΔBV was only analyzable for 64 patients at PDminus1 and 62 patients at PD, which could explain the low AUC in the ROC-analyses. Furthermore, it illustrates that DCE-CT is a demanding technique, which may limit the translation of DCE-CT from a research tool to a clinial tool. Fourthly, there is a risk of introducing target lesion selection bias, due to the relative short Z-axis of DCE-CT (8 cm). A single target lesion was chosen, but this lesion was not necessarily representative of other target lesions in the same patient due to intertumoral heterogenicity^29^. A fifth limitation is that the clinical decision making based on RECIST1.1 in this study represents a bias, as RECIST v1.1 may not nessearily identify PD at the true time point. Finally, a cut-off value could not be defined in this study, limiting the clinical utility of these parameters in daily clinical practice.

## Conclusions

In conclusion, DCE-CT identified BV is a new image-based biomarker of therapy progression in patients with mRCC.

## Data Availability

The datasets generated and/or analyzed during the current study are not publicly available due to further analysis of data for upcoming publications, but are available from the corresponding author on reasonable request.
